# Axon reflex-mediated vasodilation is reduced in proportion to disease severity in familial amyloid polyneuropathy

**DOI:** 10.1186/1750-1172-10-S1-P57

**Published:** 2015-11-02

**Authors:** Thierry Kuntzer, Irène Calero-Romero, Bernard Waeber, Francois Feihl

**Affiliations:** 1 Lausanne University Hospital CHUV, Department of Clinical Neurosciences, 1011, Lausanne, Switzerland; 2 Lausanne University Hospital CHUV, Division of Clinical Pathophysiology, 1011, Lausanne, Switzerland

## Background

Axon reflex-mediated neurogenic cutaneous vasodilatation in response to histamine reflects C-fibre dysfunction. We aimed to evaluate the distribution of the vascular flare area measured by laser doppler imaging (“LDIflare area”) in familial amyloid polyneuropathy (FAP) and in healthy volunteers.

## Methods

Twenty-one subjects were contacted to participate in this study. Eleven FAP patients and 10 normal volunteers were recruited for this hospital's ethics committee approved study. Confirmed genetically FAP patients were recruited from our registry, and were prospectively re-examined to determine the frequency of neuropathic motor, sensory, and autonomic symptoms and findings, to measure nerve conduction parameters, and to assess thermal testing performed using a Medoc TSA-II thermal analyzer. LDIflare areas were induced by iontophoresis (at 100uA for 1 min) of histamine at the forearm and at the leg.

## Results

Six patients had a FAP of variable severity, one had a generalized analgesia secondary to leprosy (used as positive control). Four patients had to be excluded from the analysis as it was not possible to quantify neuropathy severity (asymptomatic carriers). All but one had a Val30Met mutated TTR gene. The median neurological impairment score of the lower limbs (NIS_LL) was 9.3 (0 to 27). Half of the patients had reduced or absent sural nerve potential. The warmth detection thresholds in the feet were higher in patients group (ctrl=37.6°C, 35.3 to 38.3: patients=42.2°C, 38.2 to 48.8: p<0.015), indicating small fiber impairment.

Compared to controls, patients had lower LDIflare areas in the legs (ctrl=13.0, 9.2 to 19.8cm2: patients=5.8, 2.7 to 11.4cm2) and in the forearms (ctrl=21.7, 14.3 to 26.1cm2: patients=12.4, 4.2 to 20.6 cm2). However, the differences were statistically different for the legs only (p=0.015) (p=0.089 for the arms). There was an excellent correlation between the degree of LDIflare area and NIS_LL (Spearman's rank correlation for forearm; r = -0.71, p<0.074: for leg; r = -0.84, p<0.019).

## Conclusion

Our study underscores that in FAP patients, the amount of LDIflare area is reduced in proportion to disease severity.

